# Packing Densification Response–Constrained Fractal Characterization and Compaction Performance Evaluation of Widely Graded Granular Materials

**DOI:** 10.3390/ma19122675

**Published:** 2026-06-22

**Authors:** Guo-Feng Ren, Xin-Qing Wang, Yi Wang, Qiu-Yue Hu, Xiang-Jun Pei, Xiao-Chao Zhang

**Affiliations:** 1 Sichuan Water Development Investigation, Design & Research Co., Ltd., Chengdu 610213, China; 2 Department of Architecture and Civil Engineering, Sichuan Jiaotong Polytechnic University, Chengdu 611000, China; 3 State Key Laboratory of Geohazard Prevention and Geoenvironment Protection, Chengdu University of Technology, Chengdu 610059, China; 4 Tianfu Yongxing Laboratory, Chengdu 610213, China

**Keywords:** widely graded granular materials, particle-size distribution, fractal characterization, packing densification response, effective particle-size range, maximum dry density

## Abstract

**Highlights:**

- A response-constrained PSD truncation framework is proposed for non-ideal PSDs.
- The unit-mass densification contribution coefficient identifies effective size ranges.
- A preliminary lower fitting limit is supported by geometry and response data.
- Truncated indices improve maximum dry density characterization for non-ideal PSDs.

**Abstract:**

Not all particle-size fractions in widely graded granular materials contribute equally to compaction densification. For non-ideal particle-size distributions (PSDs) with local deviations or fine-end disturbances, the full-range fractal index may be influenced by particle-size fractions that contribute weakly to densification and, therefore, may not consistently represent the maximum dry density response. To address this problem, this study proposes a response-constrained truncation framework to identify a more effective PSD fitting range for fractal characterization. First, 20 concave and S-shaped PSDs from previous experiments were re-analyzed to compare full-range and truncated indices. Then, 21 progressively truncated specimens derived from three standard fractal PSDs were tested by relative density experiments. A unit-mass densification contribution coefficient, η_j_, was defined from adjacent maximum dry density differences and particle-fraction mass contents. The η_j_-d responses exhibited unimodal patterns, and the transition diameter d_c_ shifted with PSD coarseness. For the two material sources, replacing the full-range index with the truncated index increased the R^2^ values between the fractal index and maximum dry density from 0.195 to 0.886 and from 0.191 to 0.856, respectively. A continuous percentile search showed that the optimal characteristic scale was concentrated near q ≈ 30, with a robust common optimum of q = 30.53. Sensitivity analysis for β = 0.85–0.95 indicated that 0.225d_30_ falls within the transition region from highly effective filling to reduced densification efficiency. Accordingly, d_L_ = 0.225d_30_ is proposed as a preliminary engineering estimate of the lower fitting limit for non-ideal PSDs. The framework is intended for widely graded materials whose full-range fractal parameters are inconsistent with compaction response.

## 1. Introduction

The compaction performance of engineering granular materials is closely related to particle-size distribution (PSD), particle morphology, and packing structure [1,2,3,4,5,6,7,8,9,10]. Concrete aggregates, mine waste rock, tunnel spoil, rockfill, and coarse-grained fill materials are typically polydisperse systems. Their macroscopic dense state is jointly controlled by skeleton formation, void filling, and particle interference [11,12,13,14]. For widely graded materials, a PSD descriptor that reflects the effective packing structure is essential for compaction evaluation and gradation design [15,16].

From this perspective, different particle-size fractions can be understood as playing different roles during densification. Early studies by Westman and Hugill, Furnas, and McGeary clarified the densification role of small particles filling the voids between larger particles [1,2,3]. The Andreasen–Andersen continuous gradation model, the Dinger–Funk model, the compressible packing model of de Larrard, and the particle-mixture models of Yu and Standish further show that gradation continuity, size ratio, volume fraction, and particle shape jointly influence packing density and pore structure [4,5,6,7,8,9]. These studies indicate that the performance effect of PSD is structurally dependent and cannot be fully represented by a few conventional gradation indices [17,18,19,20].

Fractal models provide a compact way to describe widely graded PSDs and have been widely used for coarse-grained soils, rockfill, waste rock, and soil–rock mixtures [21,22,23,24,25,26,27,28,29,30]. However, practical PSDs often deviate from a single power-law distribution. Fine-end enrichment, local bulges in intermediate size ranges, gap gradation, and sieving uncertainty may alter the slope of a full-range log–log regression [31,32]. As a result, a single fractal index may simultaneously contain information from skeleton particles, effective filling particles, and non-structural fractions, weakening its relationship with compaction performance [32].

This study does not imply that all widely graded PSDs should be truncated. For smooth and continuous PSDs close to a single power law, a full-range fractal index may still be appropriate. The focus here is on a more common engineering case: non-ideal PSDs with local deviations or fine-end disturbances, where the same nominal fractal index may correspond to different curve shapes and different compaction responses. In such cases, the full-range index may not represent the effective particle-size interval that controls packing densification.

These packing theories also imply that different particle-size fractions should not be treated as equally effective in densification. However, conventional full-range fractal fitting gives all size ranges statistical influence in the log–log regression, even when some fine-end or locally deviated fractions contribute weakly to the compaction response. This creates a gap between PSD mathematical fitting and packing-response characterization. Therefore, a response-based criterion is needed to identify the PSD interval that better represents the effective packing structure.

Based on this understanding, larger and skeleton particles form the main contact framework, and intermediate particles may enter skeleton voids and enhance densification, whereas very fine particles may fill micro-voids but do not necessarily contribute proportionally to skeleton rearrangement. Therefore, the fractal fitting of non-ideal PSDs should not mechanically include all size fractions; instead, an effective lower fitting limit related to packing structure and compaction response should be identified.

The main contributions of this study are as follows: (1) a unit-mass packing densification contribution coefficient, η_j_, is defined to quantify the normalized contribution of each particle-size interval to maximum dry density; (2) a transition diameter, d_c_, is identified from the unimodal η_j_-d response, revealing the migration of the effective filling size with PSD coarseness; and (3) a geometrically and response-constrained lower fitting limit, d_L_ = 0.225d_30_, is proposed as an engineering estimate for fractal fitting of non-ideal PSDs.

## 2. Materials and Methods

### 2.1. Fractal PSD Model and Full-Range Fitting Bias

For a continuous PSD with maximum particle size dmax, the cumulative passing percentage P(d) can be expressed using a Talbot-type power function or fractal form:

(1) $$P(d)={\left(d/d_{max}\right)}^{n},$$

where n is the gradation index. In a mass-based particle-size fractal model, the fractal dimension D commonly satisfies D = 3 − n. Equation (1) provides a single-parameter representation of PSD coarseness, but it assumes that all particle-size intervals approximately follow the same scale law. When local deviations exist, the full-range fitted n may include both the dominant packing structure and non-power-law disturbances.

For widely graded granular materials, even a small amount of fine particles may strongly influence the log–log regression slope because of the large size span. If these particles mainly occupy free-filling or micro-void states, their statistical influence on the fractal index may exceed their actual contribution to skeleton packing. Therefore, response-constrained truncation is needed for non-ideal PSDs to reduce the leverage of low-efficiency fine-end fractions.

### 2.2. Skeleton-Filling-Interference Roles in Polydisperse Granular Systems

**Figure 1:**
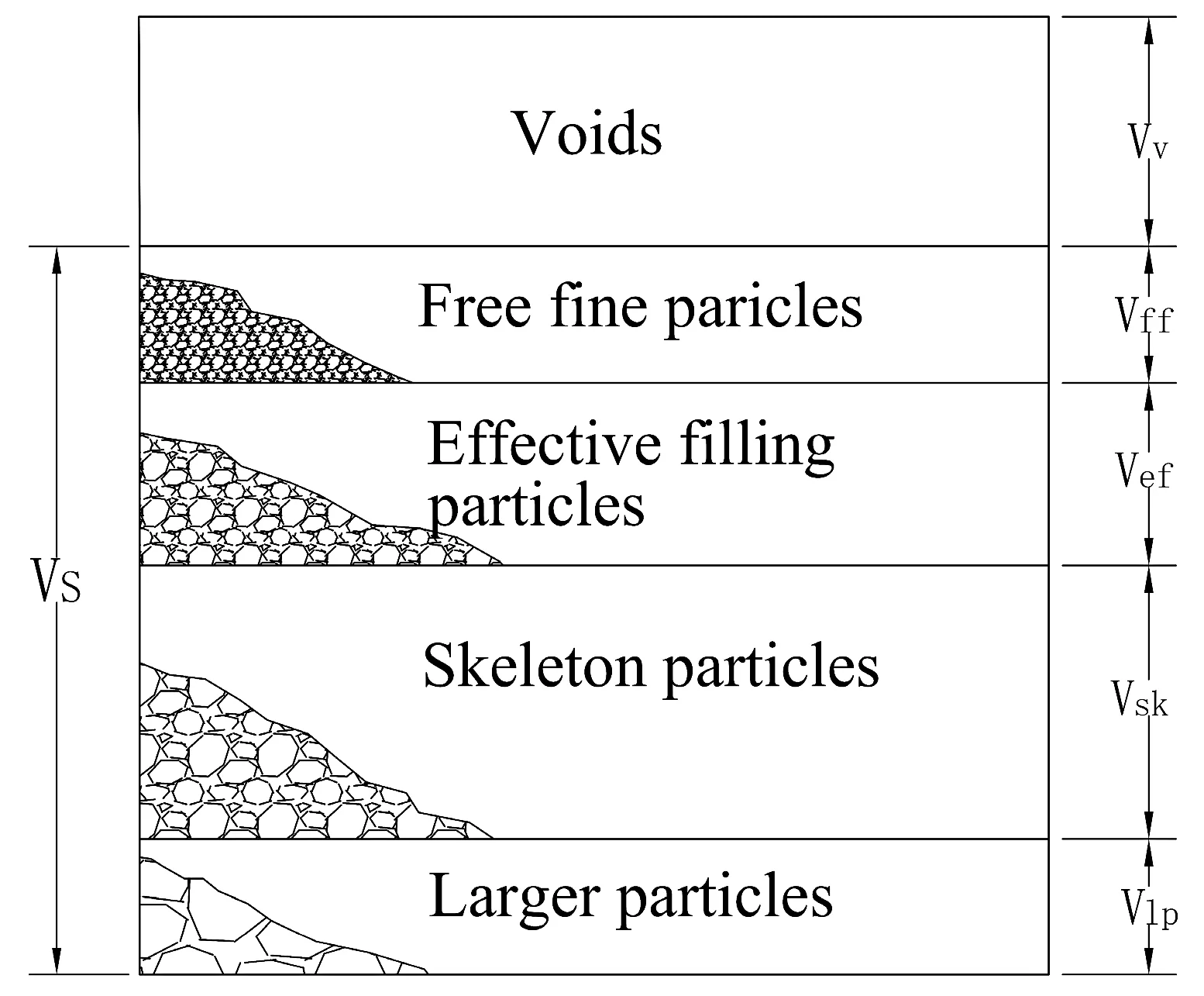
Conceptual model of the functional partition of particle fractions in widely graded granular materials.

The densification of a polydisperse granular system can be conceptualized as a combination of skeleton formation, pore filling, and interference-induced rearrangement. In this study, particle fractions are divided into larger particles, skeleton particles, effective filling particles, and free fine particles according to their packing functions. Larger particles and skeleton particles form the main load-bearing framework through contact networks. Effective filling particles enter skeleton voids and participate in local rearrangement during vibration or compaction. Free fine particles mainly occupy micro-voids or pore ends, and their variation is more likely to be reflected as a change in the PSD tail. This conceptual partition is illustrated in Figure 1.

This partition is used here as a conceptual interpretation of packing functions rather than as a directly measured contact-network classification. The quantitative boundary between effective and less effective fractions is not prescribed a priori but is inferred from the subsequent η_j_-d response and the transition diameter d_c_.

This functional partition means that the effective PSD fitting range should include skeleton particles and effective filling particles but should not be dominated by free fine particles. Truncation does not deny the filling role of fine particles; instead, it shifts the fitting emphasis toward the size interval that better reflects the dominant packing structure and compaction response.

### 2.3. Implications of Classical Packing Models for Effective Particle-Size Ranges

The Andreasen–Andersen model emphasizes the role of a continuous size distribution in high-density packing [4]. The Dinger–Funk model introduces a minimum size term to account for finite PSD ranges [5]. The linear packing density model and the compressible packing model describe filling, loosening, and wall effects among particles of different sizes [6,33]. The Yu–Standish series of models incorporates size distribution, non-spherical particles, and multicomponent mixtures into porosity prediction [7,8,9].

These models show that PSD does not affect packing density through equal weighting of all size fractions. Instead, the effect depends on geometric role, size ratio, and volume fraction. The response-constrained truncation method proposed here is consistent with this physical basis, but the objective differs: classical packing models often predict absolute packing density, whereas this study uses compaction response to identify a physically meaningful effective size interval for fractal PSD fitting.

### 2.4. Tetrahedral Void Scale and Candidate Truncation Ratio

**Figure 2:**
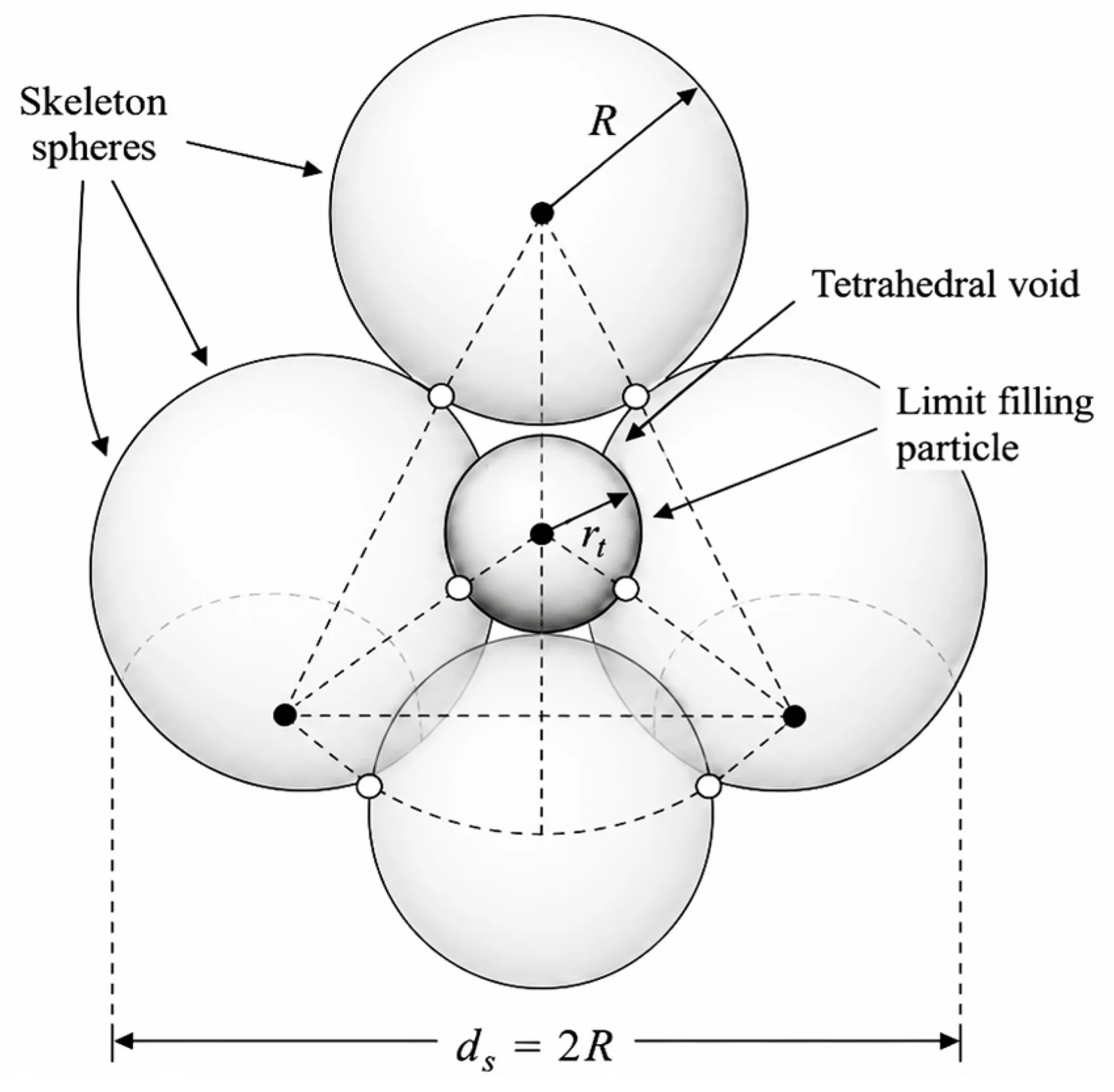
Geometric derivation of the 0.225 diameter ratio from a tetrahedral void formed by four equal skeleton spheres. R and r_t_ denote the radii of the skeleton sphere and the limit filling particle, respectively.

In face-centered cubic or hexagonal close packing of equal spheres, the tetrahedral void formed by four mutually tangent equal skeleton spheres is one of the smallest basic voids. The geometric relation is illustrated in Figure 2.

If the radius of the skeleton sphere is R, the radius r_t_ of the small sphere that can be accommodated in a tetrahedral void is given by the following:

(2) $$\frac{r_{t}}{R}=\frac{\sqrt{6}}{2}-1\approx 0.225,$$

and because the diameter ratio equals the radius ratio,

(3) $$\frac{d_{t}}{d_{s}}=\frac{r_{t}}{R}=\frac{\sqrt{6}}{2}-1\approx 0.225$$

The coefficient 0.225 is not an empirical fitting coefficient of this study. It is the classical geometric ratio for a limiting particle accommodated in a tetrahedral void of equal-sphere close packing. In a continuous PSD, however, no unique skeleton diameter d_s_ exists. Therefore, a PSD characteristic scale that is consistent with compaction response must be identified. To avoid relying on a limited set of discrete candidate sizes, a continuous percentile diameter d_q_ is introduced and searched over the percentile space q to match d_c_.

It should be noted that this ratio is derived from ideal equal-sphere packing and should not be interpreted as the exact pore-throat size of real angular particles. Particle angularity, elongation, surface roughness, and breakage may alter the local void geometry. Therefore, the tetrahedral ratio is used only as a physically interpretable geometric reference, and its relevance is further examined using the η_j_-d response and percentile search. Alternative assumptions, such as octahedral voids or cubic packing, would correspond to larger void scales than the tetrahedral case and may, therefore, lead to larger candidate filling or truncation diameters. These packing geometries are not used to define d_L_ in this study, but they may be considered in future work when comparing different geometric reference scales.

### 2.5. Truncated-Domain Fractal PSD Equation

The lower fitting limit is denoted by d_L_. For d ≥ d_L_, the normalized truncated-domain fractal PSD can be written as follows:

(4) $$P_{t\left(d\right)}=\frac{d^{\left\{3-D_{t}\right\}}-d_{L}^{\left\{3-D_{t}\right\}}}{d_{max}^{\left\{3-D_{t}\right\}}-d_{L}^{\left\{3-D_{t}\right\}}}$$

where D_t_ is the fractal dimension in the truncated domain and Pt(d) is the normalized cumulative passing percentage within the truncated interval. Compared with full-range fitting, Equation (4) explicitly includes d_L_, shifting the key issue from fitting the PSD to determining a physically reasonable lower fitting limit.

### 2.6. Data Sources, PSD Design, and Progressive Truncation Tests

**Figure 3:**
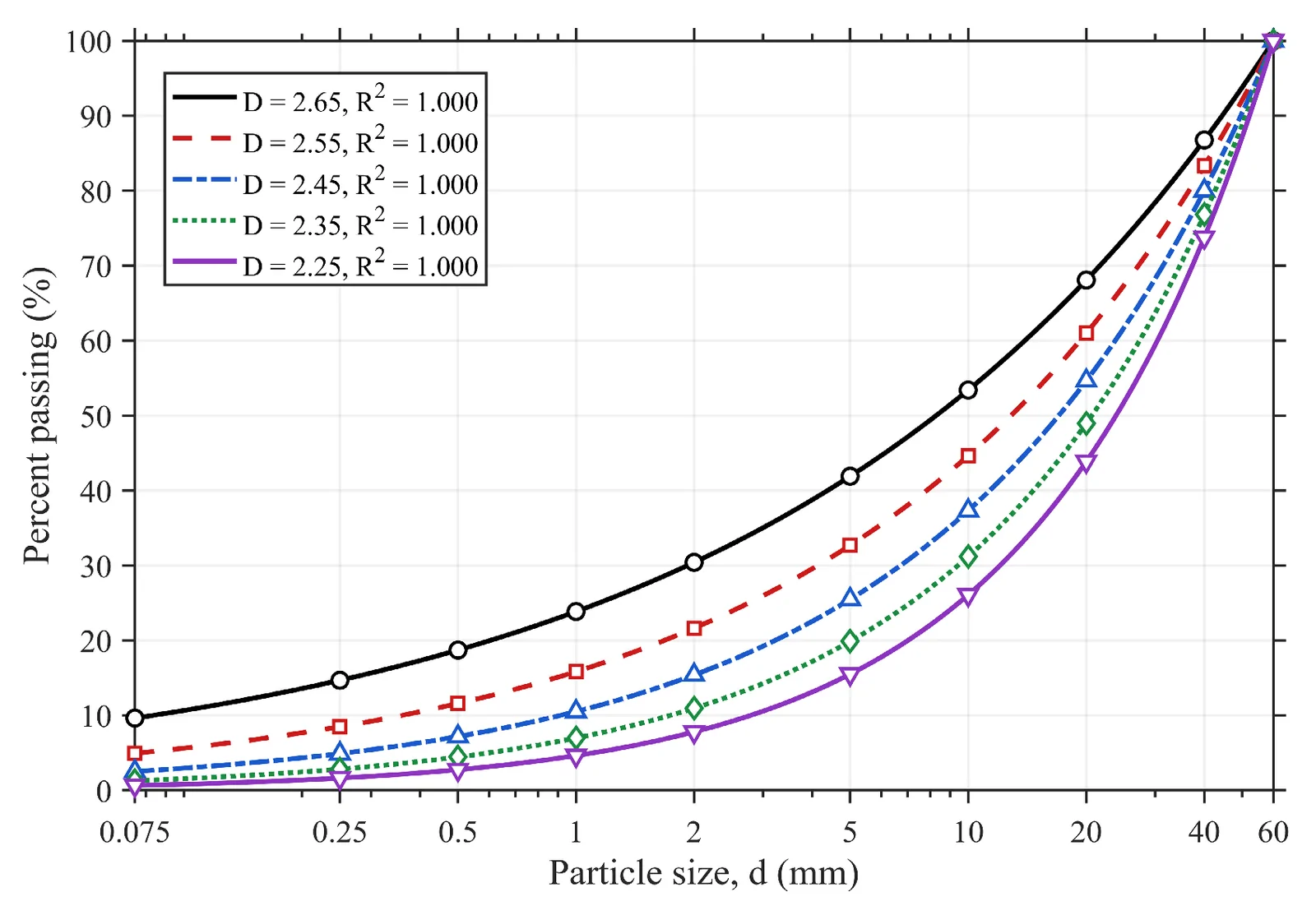
Gradation curves of concave PSDs.

**Figure 4:**
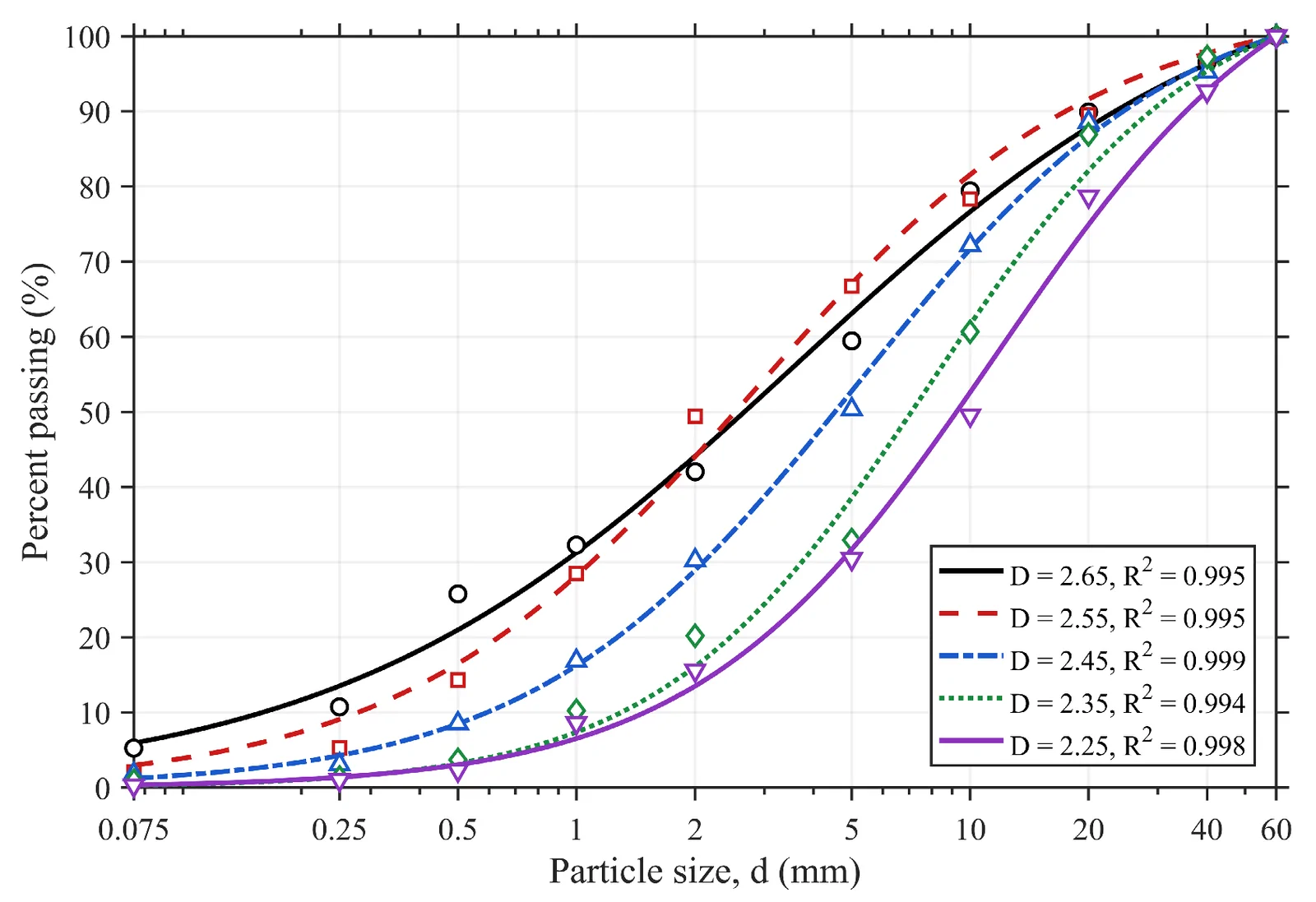
Gradation curves of S-shaped PSDs.

**Figure 5:**
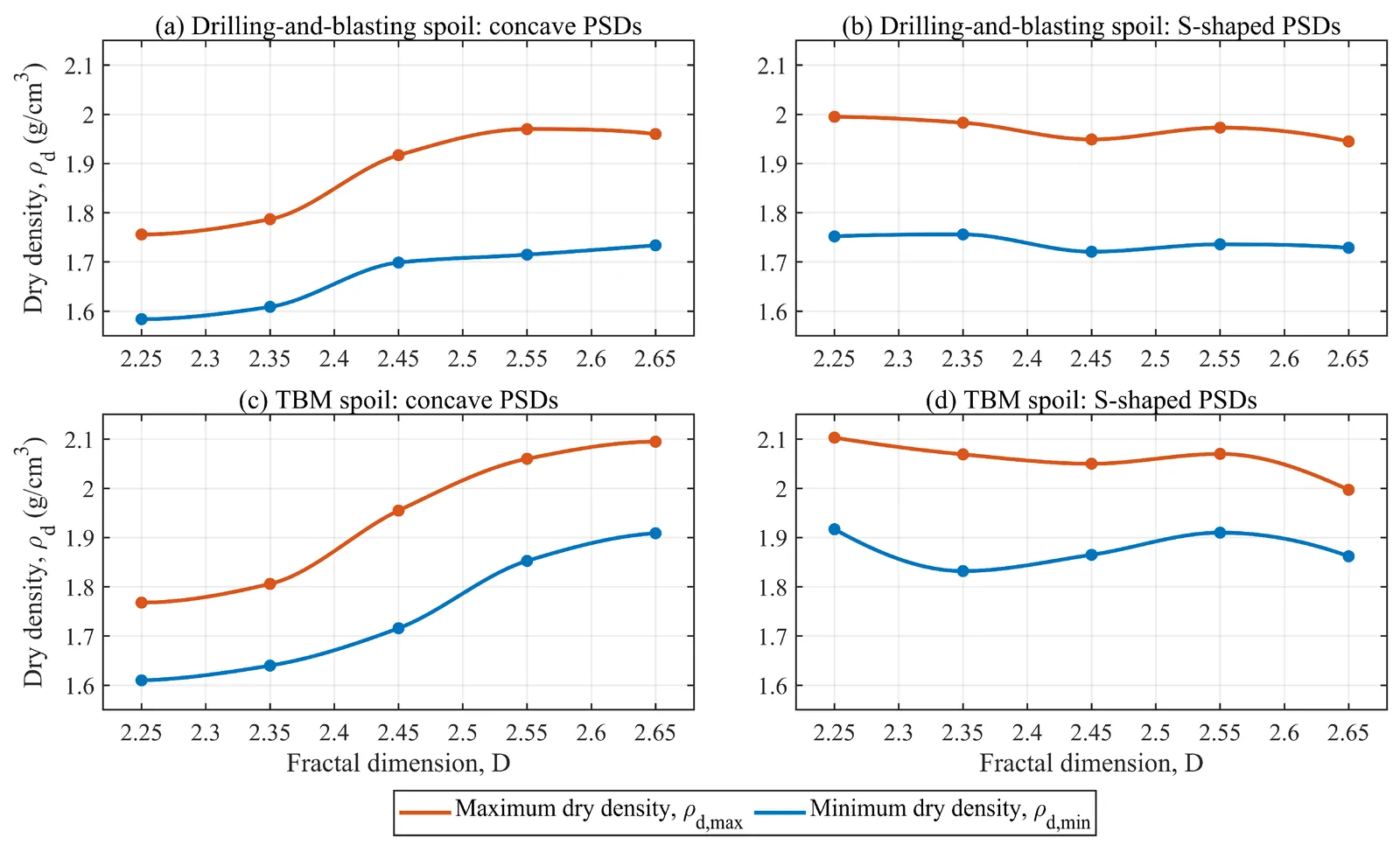
Variations in maximum and minimum dry densities of drilling-and-blasting and TBM spoils under concave and S-shaped PSDs. Panels show concave and S-shaped PSDs for the two material sources.

Two types of PSD data were used. The first type consists of 20 concave and S-shaped PSDs obtained in a previous study, where 10 designed PSDs were tested using drilling-and-blasting spoil and TBM spoil. These data were previously used to establish a gradation equation and a maximum dry density prediction model [34]. In the present study, the same data are re-analyzed to examine how full-range and truncated fractal indices characterize non-ideal PSDs. They are used as an internal consistency and parameter-reconstruction dataset rather than as independent external validation data. Figure 3 and Figure 4 show representative concave and S-shaped PSD curves from this dataset, and Figure 5 shows the corresponding extreme dry density responses.

The second type consists of three standard fractal parent PSDs, denoted as Groups A, B, and C, with n = 0.35, 0.55, and 0.75, respectively. Each group includes one parent PSD and six progressively truncated PSDs, giving 21 specimens in total. The maximum and minimum dry densities were measured using a coarse-grained soil relative density apparatus (ZKM-301A, Sichuan Ruihong Technology Co., Ltd., Chengdu, Sichuan, China) according to ASTM D4253/D4253M-16e1, ASTM D4254-16 and GB/T 50123-2019 [35,36,37]. The maximum dry density was used to calculate η_j_, and the minimum dry density was used to help describe the loose packing state.

The characteristic particle sizes of the three parent PSDs are listed in Table 1.

The complete gradation compositions of the progressively truncated specimens are provided in Supplementary Table S1. These three parent PSDs were selected to represent typical fine-to-coarse fractal gradation states within the tested size range, rather than to cover all possible engineering PSDs.

The first dataset was used as a re-analysis and internal consistency dataset, whereas the progressive truncation tests of Groups A–C were used to construct the η_j_-d response and identify d_c_.

### 2.7. Unit-Mass Packing Densification Contribution Coefficient

Progressive truncation provides the maximum dry density difference between two adjacent PSDs. Because different particle-size intervals have different mass fractions, a direct comparison of density increments would be affected by particle-fraction content. To eliminate this effect, the unit-mass packing densification contribution coefficient η_j_ is defined as follows:

(5) $$\eta_{j}=\frac{\Delta \rho_{j}}{p_{j}}=\frac{\rho_{d,max}(s_{j})-\rho_{d,max}(s_{j+1})}{p_{j}}$$

where S_j_ and S_j+1_ are two adjacent truncated PSDs; ρ_d,max_(S_j_) and ρ_d,max_(S_j+1_) are their maximum dry densities; Δρ_j_ is the maximum dry density difference; and p_j_ is the mass fraction of the corresponding particle-size interval. The mass fraction p_j_ is calculated from the cumulative passing percentage:

(6) $$p_{j}=\frac{P_{\left(d_{right}\right)}-P_{\left(d_{left}\right)}}{100}$$

The use of Δ*ρ*_j_ alone would mix the densification effect of a fraction with its mass content. By normalizing Δ*ρ*_j_ with p_j_, η_j_ converts the density change into a unit-mass contribution, allowing different particle-size intervals to be compared on the same basis. Therefore, η_j_ should be regarded as a response-normalized index for densification efficiency rather than as an intrinsic material constant. A larger η_j_ indicates a stronger unit-mass contribution to densification. It should be emphasized that η_j_ is not a directly measured dry density point; it is a response index calculated from adjacent maximum dry density differences and interval mass fractions.

The particle-size intervals, representative diameters, and corresponding mass fractions used for η_j_-d fitting are summarized in Table 2.

The discrete η_j_ values used for constructing the η_j_-d response curves are given in Table 3.

### 2.8. Identification of the Packing-Response Transition Diameter d_c_

After constructing the unimodal response curve from the η_j_-d data, the response generally exhibits an ascending stage, a peak region, and a descending stage. The ascending stage indicates that particles gradually enter a more effective void-filling state; the peak region indicates high unit-mass densification contribution; and the descending stage implies that the filling efficiency decreases and that larger particles may begin to interfere with the existing skeleton arrangement.

The transition diameter d_c_ is defined as the first point on the descending branch of the unimodal fitted curve where η(d) decreases to βη_max_:

η(d_c_) = βη_max_, β = 0.90, (7)

where η_max_ is the peak value of the unimodal fitted curve. The value β = 0.90 was selected to identify a near-peak transition zone rather than the exact peak position. When η decreases to 0.90η_max_ on the post-peak branch, the unit-mass densification contribution has clearly departed from the peak level but remains within the high-efficiency neighborhood. Therefore, d_c_ is used to represent the transition from highly effective filling to reduced densification efficiency. To examine whether this interpretation depends strongly on a single threshold, β = 0.85, 0.90, and 0.95 were further considered in the sensitivity analysis.

The discrete η_j_ values in Table 3 were calculated from the maximum dry density differences between two adjacent progressively truncated specimens and, therefore, may contain local point-to-point fluctuations. To extract the dominant response trend, the η_j_–d_j_ data of each group were first transformed into η_j_–ln d_j_ space. A shape-preserving piecewise cubic interpolation was then combined with a unimodal constraint to construct a continuous response curve with one dominant peak. The peak value η_max_ and the corresponding peak location were obtained from the continuous curve. The transition diameter d_c_ was then determined by interpolating the descending branch of this curve at η(d) = βη_max_. The same curve-construction and interpolation procedure was applied to all three groups. Because only six discrete η_j_ points were available for each group, this procedure was used to identify the dominant response trend and the transition region, rather than to establish a universal analytical response model for all PSDs. The shape-preserving piecewise cubic interpolation, unimodal-constrained curve construction, sensitivity analysis, and figure preparation were performed using MATLAB R2021b (MathWorks, Natick, MA, USA).

## 3. Results

### 3.1. Parameter Reconstruction Before and After PSD Truncation

For concave PSDs, which are close to single power-law distributions, the effective gradation index n_t_ obtained after truncation with d_L_ = 0.225d_30_ remained nearly identical to the original n. For S-shaped PSDs, where the fine end and intermediate size range contain local deviations, n_t_ became markedly lower than the nominal full-range n. To maintain comparability with the full-range Talbot index, the PSD segment d ≥ d_L_ was re-normalized and fitted with the truncated-domain equation.

Table 4 shows that truncation does not artificially alter PSDs that already follow a power law. Instead, it mainly corrects the representation of PSDs with non-structural local deviations. For S-shaped PSDs, n_t_ changed from the nominal range of n = 0.35–0.75 to n_t_ = 0.306–0.485, which reopens the structural distinction between PSDs that share the same nominal index.

To further evaluate whether truncation improves the characterization of compaction response, the measured maximum dry densities of the 20 concave and S-shaped PSDs were correlated with both the full-range index n and the truncated effective index n_t_. A quadratic fit was used for each material source. The quadratic fit was used here as a simple and identical comparison form for both n and n_t_, rather than as an optimized prediction model. The purpose was to compare the relative ability of the full-range and truncated indices to characterize maximum dry density response under the same fitting form.

**Figure 6:**
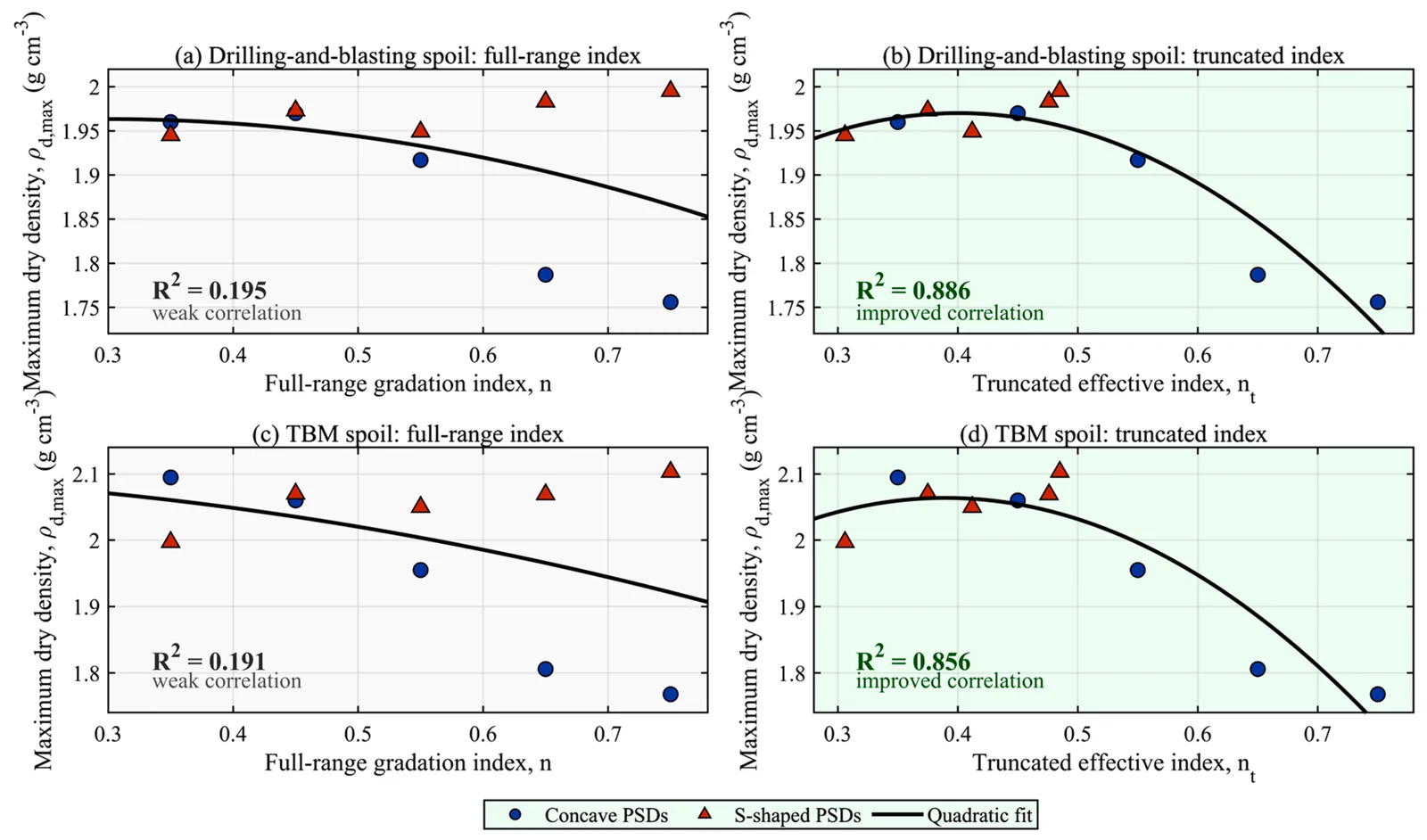
Comparison of maximum dry density characterization before and after PSD truncation.

As shown in Figure 6, the relationship between ρ_d,max_ and the full-range index n was weak, with R^2^ = 0.195 for drilling-and-blasting spoil and R^2^ = 0.191 for TBM spoil. After replacing n with n_t_, the corresponding R^2^ values increased to 0.886 and 0.856, respectively. Because the available datasets were not designed as repeated statistical experiments, this R^2^ comparison is used as internal consistency evidence rather than as a formal statistical significance test. This indicates that response-constrained truncation improves the ability of the fractal index to characterize the maximum dry density response of non-ideal PSDs.

**Figure 7:**
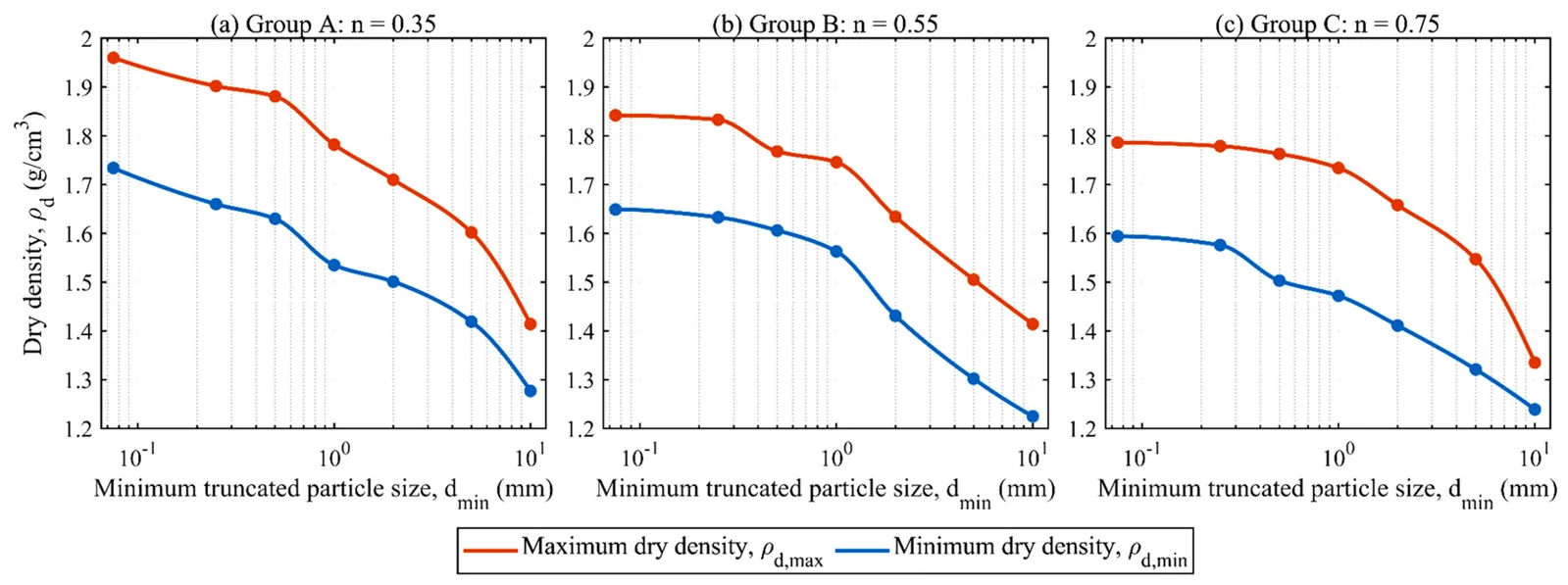
Variations in maximum and minimum dry densities of Groups A–C with increasing minimum truncated particle size.

The progressive truncation tests of Groups A–C were then used to quantify the density response caused by the stepwise removal of fine-size fractions. As shown in Figure 7, both ρ_d,max_ and ρ_d,min_ generally decreased as the minimum truncated particle size increased from 0.075 to 10 mm. This trend shows that fine fractions contribute to void filling and packing densification, and that different particle-size intervals contribute unequally to compaction density. These observations provide the experimental basis for defining the unit-mass packing densification contribution coefficient η_j_ from the maximum dry density differences between adjacent truncated PSDs.

This re-analysis of the previous PSDs and the progressive truncation tests together support the need for a response-constrained effective fitting interval. They also motivate the construction of η_j_-d response curves to identify the compaction-response transition diameter d_c_.

### 3.2. Density-Response Basis for η_j_ Calculation

The progressive truncation tests provide the density-response basis for calculating η_j_. For each adjacent pair of truncated PSDs, the maximum dry density difference Δρ_j_ was normalized by the corresponding interval mass fraction p_j_ to obtain η_j_. Therefore, η_j_ should be interpreted as a response-normalized index derived from the measured density sequence, rather than as an independently measured density parameter. The purpose of introducing η_j_ is not to reconstruct the density sequence itself, but to compare the relative unit-mass densification contribution of different particle-size intervals and to support the subsequent identification of the η_j_-d response trend and transition diameter d_c_.

### 3.3. Unimodal Response of η_j_ to Particle Size

**Figure 8:**
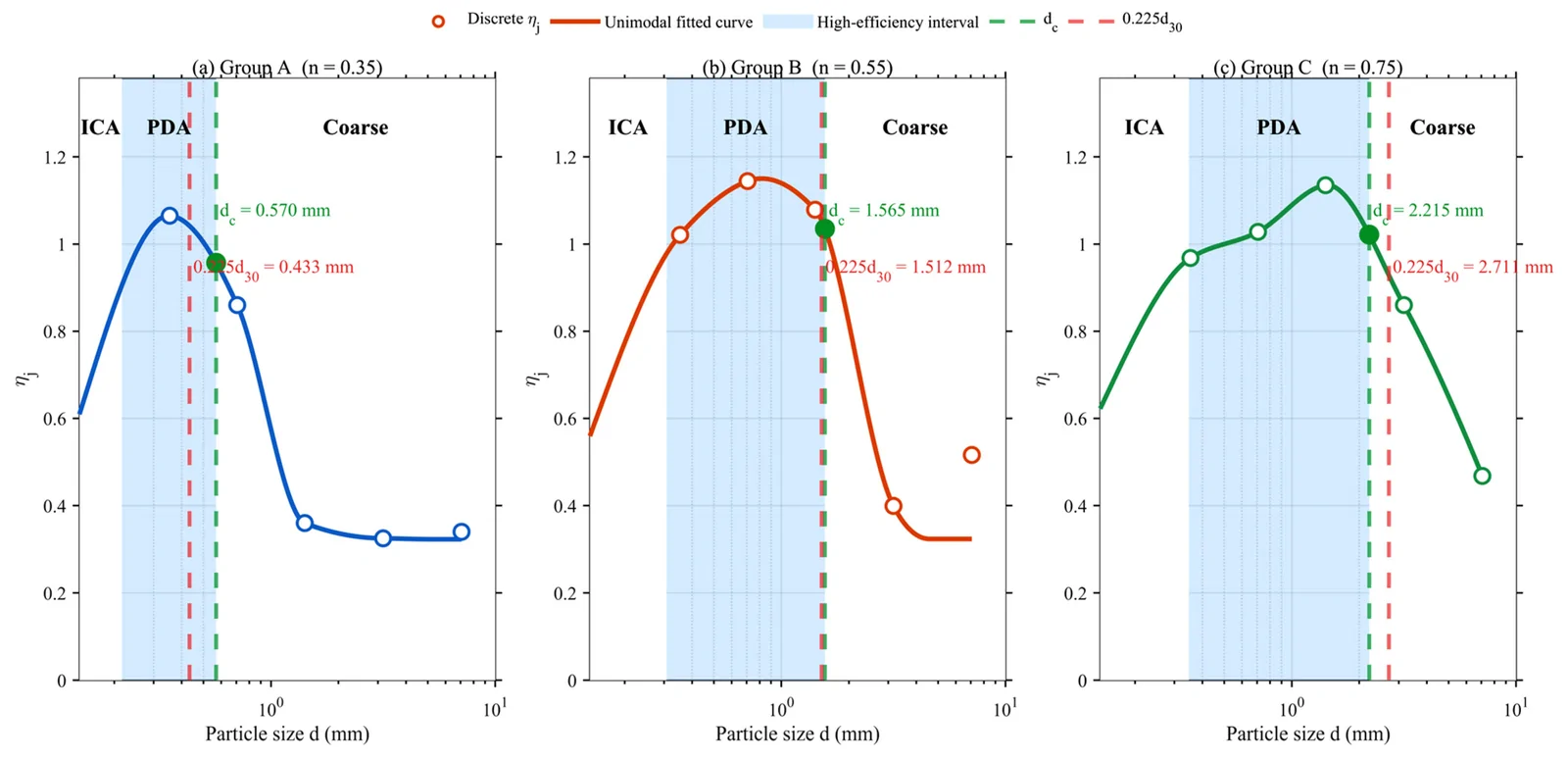
Unimodal response of the unit-mass packing densification contribution coefficient ηj to particle size for Groups A–C. Note: Symbols denote the discrete ηj values listed in Table 3; solid curves denote the unimodal-constrained response curves; and shaded areas denote the high-efficiency intervals.

As shown in Figure 8, the η_j_-d curves of Groups A–C exhibit clear unimodal responses, indicating that the unit-mass densification contribution first increases from the fine-particle range to an effective filling range and then decreases as particle size further increases. With increasing PSD coarseness, both the peak position and the high-efficiency interval shift toward larger particle sizes. In Group A, the high-efficiency interval is mainly around 0.25–0.5 mm; in Group B, it shifts to approximately 0.5–2 mm; and in Group C, it extends further toward larger particle-size intervals. This migration suggests that the effective filling scale is controlled by the characteristic skeleton-pore size rather than by a fixed particle diameter. Therefore, using a constant lower truncation size for different PSDs may either remove particles that still contribute to densification in finer PSDs or retain low-efficiency fine fractions in coarser PSDs.

### 3.4. Continuous Percentile Search and Engineering Selection of d_30_

To avoid dependence on a limited set of discrete characteristic diameters, the percentile diameter d_q_ was searched continuously. For the standard fractal PSDs of Groups A–C, Equation (1) gives the following:

d_q_ = d_max_(q/100)^1/n^. (8)

The relative error between the tetrahedral-void estimate 0.225d_q_ and the response-based transition diameter d_c_ is defined as follows:

E(q) = |0.225d_q_ − d_c_|/d_c_. (9)

The optimal characteristic scales obtained from the continuous percentile search are summarized in Table 5.

The group-specific optimal percentiles were q* = 33.03, 31.16, and 26.60 for Groups A, B, and C, respectively, all concentrated near q ≈ 30. If only the mean relative error is minimized, the common optimum is approximately q = 31.16. However, a unified engineering scale should also avoid excessive error for any individual PSD. Therefore, a robust criterion was used:

(10) $$q_{\mathrm{robust}}\;=\;\arg\;\min_{q\in (0,100)}\;\{\max[E_{A(q)},\;E_{B(q)},\;E_{C(q)}]\}.$$

**Figure 9:**
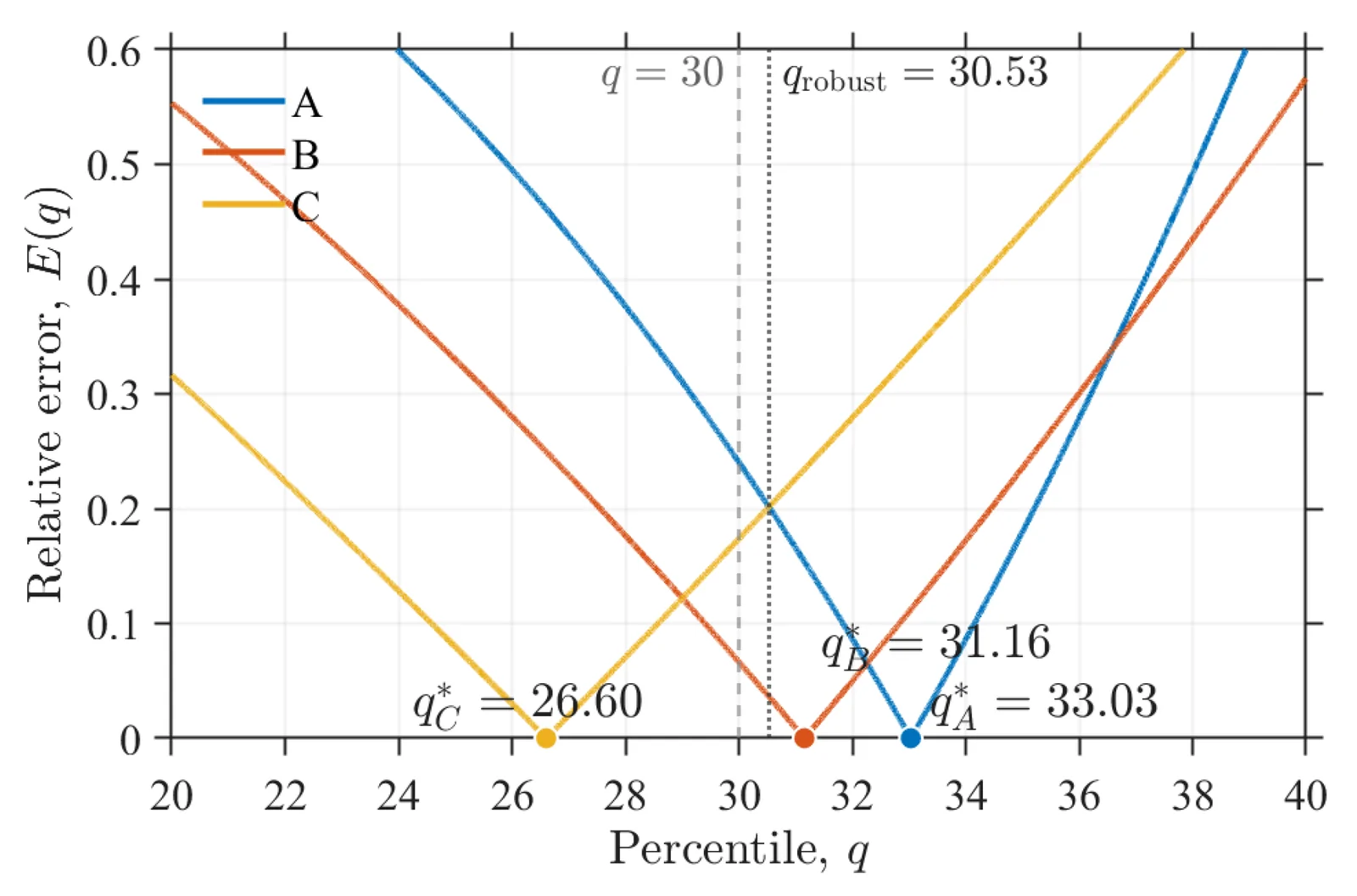
Relative error E(q) as a function of percentile q for Groups A–C. The dashed and dotted lines indicate q = 30 and q_robust_ = 30.53, respectively.

The robust optimum was identified as q_robust_ = 30.53. At this percentile, the relative errors for Groups A, B, and C were 0.202, 0.036, and 0.201, respectively. Taking E(q) ≤ 0.25 as a practical low-error criterion, the common low-error interval was q = 29.87–31.45, within which q = 30 is included. This threshold is not intended as a theoretical boundary; it is used as a practical tolerance to identify whether the geometric estimate and the response-based d_c_ fall within the same engineering scale range. Therefore, the continuous percentile search does not aim to replace the conventional d_30_ with the nonstandard d_30.53_. Instead, it verifies that d_30_ is located within a stable percentile range that is consistent with the compaction-response transition diameter d_c_. Figure 9 shows the relative error E(q) as a function of percentile q for Groups A–C.

The value q_robust_ = 30.53 should not be interpreted as a new characteristic particle-size index. Instead, it was used to verify whether the conventional d_30_ falls within a response-consistent low-error percentile interval. Therefore, the use of d_30_ is a practical engineering simplification supported by the percentile search, rather than a replacement of d_30_ by d_30.53_.

### 3.5. Sensitivity of d_c_ to the Threshold β

Because the definition of d_c_ includes the threshold parameter β, the sensitivity of the identified transition diameter to β was evaluated for β = 0.85, 0.90, and 0.95. The results are summarized in Table 6. The same curve-construction and interpolation procedure was used for all cases.

As β increases from 0.85 to 0.95, d_c_ moves toward the peak of the response curve. The scale d_L_ = 0.225d_30_ is closest to d_c_ at β = 0.95 for Group A, β = 0.90 for Group B, and β = 0.85 for Group C. This does not indicate that the optimal β changes systematically by group; instead, it indicates that 0.225d_30_ corresponds to a transition interval rather than to a single fixed-threshold point. Therefore, d_L_ = 0.225d_30_ is not obtained by tuning β but remains within the neighborhood of the transition from highly effective filling to reduced densification efficiency.

### 3.6. Proposed Lower Fitting Limit

Combining the tetrahedral void geometry, the unimodal η_j_-d response, the continuous percentile search, and the β sensitivity analysis, the following preliminary lower fitting limit is proposed for response-constrained fractal fitting of non-ideal PSDs:

d_L_ = 0.225d_30_. (11)

In Equation (11), the coefficient 0.225 originates from ideal equal-sphere tetrahedral void geometry, and d_30_ is not a strict skeleton boundary or a group-specific mathematical optimum. Rather, it is an engineering representative of the response-consistent percentile interval near q ≈ 30. Equation (11) should be interpreted as a preliminary engineering estimate for response-constrained truncation, not as a mandatory preprocessing rule for all PSDs.

## 4. Discussion

### 4.1. Mechanism of Fine-End Disturbance in Full-Range Fractal Fitting

The bias of full-range fractal fitting originates from the nearly equal statistical influence of all size points in log–log regression. Although fine fractions may have small mass contents, they may span a large logarithmic size range and, therefore, exert a leverage effect on the regression slope. For concave PSDs, all size intervals approximately follow one power law, and the fine end does not produce strong interference. For S-shaped or locally deviated PSDs, however, the fine end and intermediate segments introduce both structural and non-structural information into the full-range slope, obscuring the physical meaning of the fractal index.

A separate controlled sensitivity analysis of fines content was not conducted in this study. Therefore, the leverage effect is interpreted from the log-scale regression geometry and from the comparison between full-range and truncated indices, rather than as a statistically isolated fines-content effect.

Accordingly, the need for truncation does not depend simply on whether fine particles are present. It depends on whether the full-range parameter is inconsistent with the compaction response. For nearly power-law PSDs with stable full-range response, truncation is unnecessary. For locally deviated PSDs, response-constrained truncation helps reconstruct the meaning of the fractal parameter.

### 4.2. Differences from Existing Particle-Structure Descriptors

The proposed method differs from the dominant aggregate size range (DASR) method, the Dinger–Funk model, and fixed-sieve truncation. DASR identifies a dominant aggregate size range primarily from the perspective of coarse-aggregate contact networks [38,39]. The η_j_ method in this study starts from maximum dry density differences between adjacent truncated PSDs and identifies the effective lower fitting limit through unit-mass densification contribution. Thus, DASR emphasizes contact networks, whereas the present method emphasizes packing densification response.

In the Dinger–Funk model, the minimum particle size is a boundary parameter of a distribution function [5]. In contrast, d_L_ in this study is an effective fitting lower limit constrained by compaction response, tetrahedral void geometry, and continuous percentile search. Compared with fixed truncation at 0.075 mm, 0.5 mm, or another sieve size, d_L_ = 0.225d_30_ adapts to the skeleton scale of the PSD and is, therefore, more suitable for non-ideal, widely graded PSDs.

Therefore, the proposed method is complementary to DASR and packing-density models. DASR focuses mainly on identifying the dominant contact skeleton, and packing-density models aim to predict absolute packing density or porosity, whereas the present framework uses compaction response to identify a lower fitting limit for fractal characterization of non-ideal PSDs.

### 4.3. Reasonableness of d_30_ as an Equivalent Skeleton Scale

A real polydisperse granular system does not contain a single particle diameter that strictly defines the skeleton boundary. If only d_10_, d_20_, d_30_, d_40_, and d_50_ are compared, the result can depend on the preselected candidates. The continuous percentile search avoids this limitation by examining the match between 0.225d_q_ and d_c_ over the full percentile space.

The three group-specific optima concentrate between q = 26.60 and 33.03, and the robust common optimum is q = 30.53. Because d_30_ lies within this response-consistent interval and is already widely used in engineering gradation analysis, selecting d_30_ as the representative equivalent skeleton scale balances physical consistency and practical applicability.

### 4.4. Applicability and Boundary Conditions

The method is intended for non-ideal PSDs whose full-range fractal characterization is inconsistent with compaction response. In engineering use, non-ideal PSDs and response mismatch can be diagnosed using several indicators: systematic rather than random residuals in full-range log–log fitting, S-shaped trends, local bulges, gap gradation, fine-end enrichment, weak correlation between the full-range fractal index and maximum dry density, or obvious density-response dispersion among PSDs with similar nominal indices. These indicators are intended as practical diagnostic markers rather than fixed universal thresholds.

**Figure 10:**
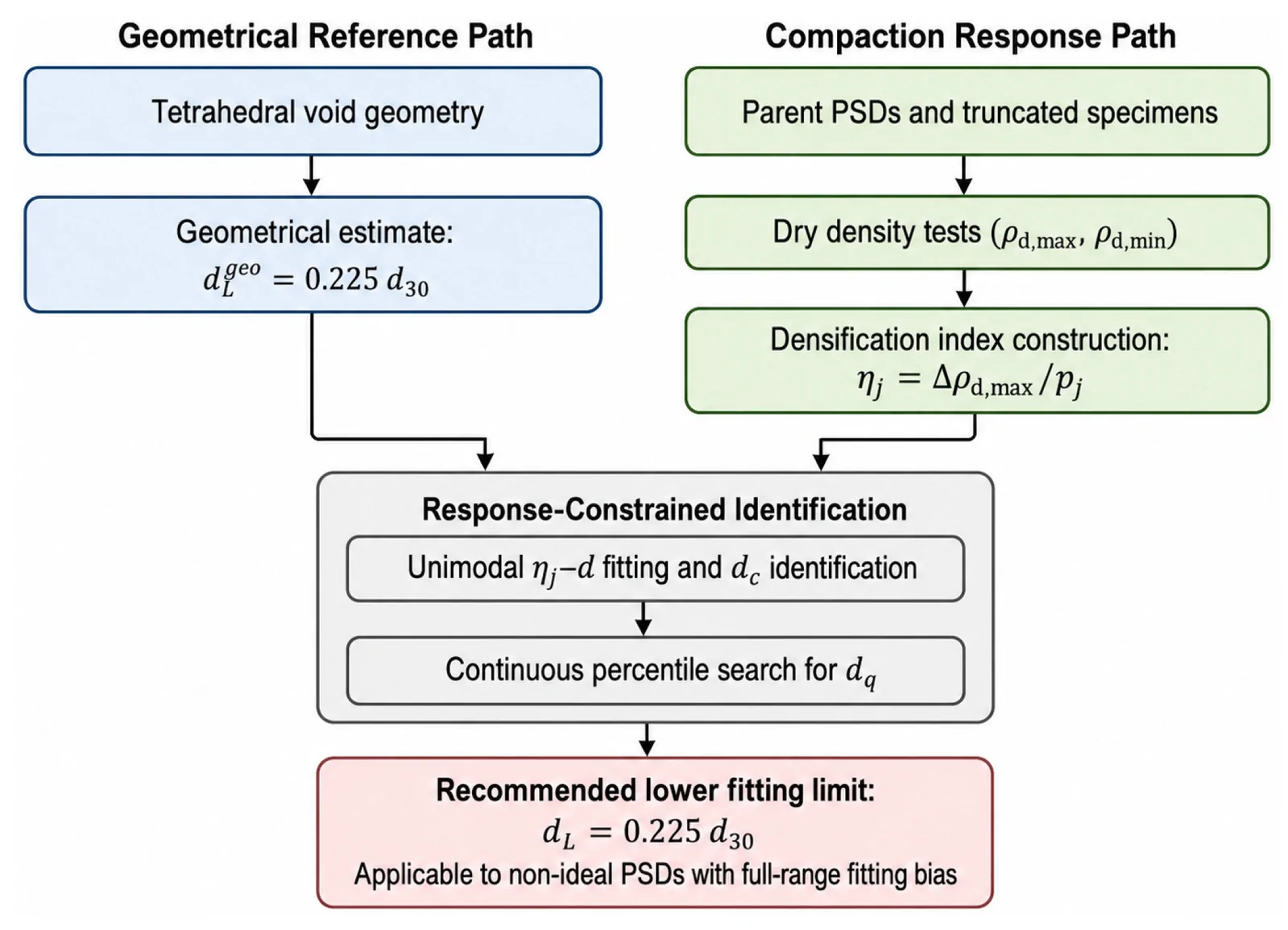
Suggested workflow for applying the response-constrained truncation framework to non-ideal PSDs.

Under such conditions, response-constrained truncation can reduce the regression influence of low-efficiency or non-structural size fractions and refocus the fractal parameter on the effective structural interval. For smooth, continuous PSDs whose full-range parameter already reflects compaction response, truncation may remove fractions that still contribute structurally. Therefore, Equation (11) should be used only after a response mismatch is identified. A suggested workflow is shown in Figure 10.

### 4.5. Implications for Structure-Performance Characterization

The proposed framework provides a response-constrained strategy for PSD characterization of non-ideal, widely graded granular materials. For powders, aggregates, waste rock, and engineering spoil, the lower fitting limit of fractal PSD analysis should not be determined only by a fixed sieve size or empirical cutoff. It should be related to the contribution of different particle-size intervals to packing and compaction.

The estimate d_L_ = 0.225d_30_ links the fitting range to both skeleton scale and densification response. The resulting fractal parameter is, therefore, more closely associated with the dominant packing structure. However, this index cannot replace strength, deformation, permeability, or durability evaluations. For regular continuous PSDs with stable full-range behavior, complete PSD information should still be retained.

### 4.6. Limitations

This study has several limitations. First, the coefficient 0.225 is derived from ideal equal-sphere close packing, whereas real particle shape, angularity, surface roughness, and breakage can alter pore-throat geometry. Therefore, Equation (11) should be understood as a geometrically referenced estimate rather than a universal constant. For highly angular crushed aggregates or materials with much larger maximum particle sizes, particle interlocking, breakage, and pore geometry may differ substantially from the idealized packing assumption. Mineral composition, surface friction, and moisture-related interparticle forces may further affect particle rearrangement and the effective filling scale. Moreover, the tetrahedral void represents only one idealized packing geometry; alternative assumptions, such as octahedral voids or cubic packing, correspond to larger void scales and would lead to larger candidate truncation diameters. A systematic comparison of different void-geometry assumptions is left for future work.

Second, the maximum particle size of the three progressive-truncation groups is 60 mm, and larger particle-size spans require further verification. Third, the unimodal curve construction of η_j_-d responses was used to extract the dominant response trend, but PSDs with dual skeletons or multiple effective filling intervals may require more flexible response models. Fourth, complete repeatability statistics were not available in the present dataset. Therefore, the R^2^ comparison, η_j_-d response analysis, and β sensitivity analysis should be interpreted as internal consistency evidence rather than complete statistical validation.

The proposed method is currently a response-constrained framework for identifying a lower fitting limit, rather than a universal predictive equation. The present dataset supports the feasibility and internal consistency of the framework, but it is not sufficient to establish d_L_ = 0.225d_30_ as a universal empirical law. Future work should test η_j_, d_c_, and d_L_ across more material types, particle shapes, particle-size spans, and datasets with complete repeatability statistics. In addition, discrete element modeling (DEM), which has been widely used to simulate interparticle contacts, contact-force transmission, and gradation-dependent packing behavior in granular assemblies [31,40,41], could be combined with the present framework in future work to examine the contact-network basis of η_j_, d_c_, and d_L_, as well as the influence of particle shape and breakage on cross-material applicability.

## 5. Conclusions

(1) The re-analysis of 20 concave and S-shaped PSDs showed that full-range fractal fitting is appropriate for nearly power-law PSDs but can be biased by fine-end or local deviations in S-shaped PSDs. After applying d_L_ = 0.225d_30_, concave PSDs remained nearly unchanged, whereas the effective indices of S-shaped PSDs changed from n = 0.35–0.75 to n_t_ = 0.306–0.485. The truncated index substantially improved the relationship with maximum dry density for both material sources.

(2) The unit-mass packing densification contribution coefficient η_j_ quantified the normalized contribution of each particle-size interval. The η_j_-d curves of Groups A–C exhibited unimodal responses, and the peak shifted toward larger particle sizes with increasing PSD coarseness. This indicates that the effective filling scale is controlled by the characteristic skeleton-pore size rather than by a fixed particle diameter.

(3) By combining tetrahedral void geometry with continuous percentile search, d_L_ = 0.225d_30_ was proposed as a preliminary engineering estimate of the lower fitting limit for non-ideal PSDs. The optimal percentiles of the three groups clustered near q ≈ 30, with a robust common optimum of 30.53. Sensitivity analysis for β = 0.85–0.95 further indicated that 0.225d_30_ lies within the transition region from highly effective filling to reduced densification efficiency.

(4) The method is mainly applicable to non-ideal PSDs with local deviations, fine-end disturbances, or a mismatch between full-range fractal parameters and compaction response. It should not be used as a mechanical preprocessing rule for smooth power-law PSDs, and its cross-material applicability requires further validation.

## Figures and Tables

**Table 1:** Characteristic particle sizes of the three parent PSDs.

Group	n	D	d_10_ (mm)	d_30_ (mm)	d_60_ (mm)	d_90_ (mm)	0.225d_30_ (mm)
A	0.35	2.65	0.083	1.924	13.941	44.404	0.433
B	0.55	2.45	0.912	6.721	23.702	49.540	1.512
C	0.75	2.25	2.785	12.050	30.364	52.136	2.711

**Table 2:** Particle-size intervals, representative diameters, and mass fractions for η_j_-d curve fitting.

j	Representative Diameter d_j_ (mm)	Particle-Size Interval (mm)	p_j (A)_	p_j (B)_	p_j (C)_
1	0.137	0.075–0.25	0.0505	0.0238	0.0098
2	0.354	0.25–0.5	0.0403	0.0228	0.0112
3	0.707	0.5–1	0.0514	0.0333	0.0188
4	1.414	1–2	0.0655	0.0488	0.0316
5	3.162	2–5	0.1150	0.1009	0.0771
6	7.071	5–10	0.1150	0.1184	0.1057

Note: The geometric mean was used because the PSD and fractal fitting are analyzed on a logarithmic particle-size scale. Arithmetic or harmonic means would shift the representative diameters toward the upper or lower bounds of each interval, respectively, but would not change the calculated η_j_ values or the main trend of unequal densification contribution among intervals.

**Table 3:** Discrete η_j_ values calculated from adjacent maximum dry density differences and interval mass fractions.

Group	η_1_	η_2_	η_3_	η_4_	η_5_	η_6_
A	0.5941	1.0650	0.8600	0.3600	0.3250	0.3400
B	0.5462	1.0211	1.1444	1.0787	0.3992	0.5161
C	0.6122	0.9680	1.0280	1.1350	0.8600	0.4680

**Table 4:** Parameter reconstruction results before and after truncation for ten PSDs.

PSD Type	Nominal D	d_30_ (mm)	d_L_ = 0.225d_30_ (mm)	Full-Range n	Truncated n_t_	Δn
Concave	2.65	1.915	0.431	0.350	0.350	0.000
Concave	2.55	4.000	0.900	0.450	0.450	0.000
Concave	2.45	6.511	1.465	0.550	0.550	0.000
Concave	2.35	9.291	2.090	0.650	0.650	0.000
Concave	2.25	11.650	2.621	0.750	0.750	0.000
S-shaped	2.65	0.784	0.176	0.350	0.306	0.044
S-shaped	2.55	1.051	0.236	0.450	0.375	0.075
S-shaped	2.45	1.974	0.444	0.550	0.412	0.138
S-shaped	2.35	4.043	0.910	0.650	0.476	0.174
S-shaped	2.25	4.869	1.096	0.750	0.485	0.265

**Table 5:** Optimal characteristic scales from the continuous percentile search.

Group	n	d_c_ (β = 0.90) (mm)	q*	d_q_* (mm)	0.225d_q_* (mm)
A	0.35	0.57	33.03	2.533	0.570
B	0.55	1.62	31.16	7.201	1.620
C	0.75	2.31	26.60	10.264	2.309

**Table 6:** Sensitivity of d_c_ and relative error of d_L_ to threshold β.

Group	d_L_ = 0.225d_30_ (mm)	d_c_ (β = 0.95) (mm)	E0.95	d_c_ (β = 0.90) (mm)	E0.90	d_c_ (β = 0.85) (mm)	E0.85
A	0.433	0.50	0.13	0.57	0.24	0.64	0.32
B	1.512	1.35	0.12	1.62	0.07	1.77	0.15
C	2.711	2.01	0.35	2.31	0.17	2.58	0.05

Note: E_β_ = |d_L_ − d_c_(β)|/d_c_(β), where E_β_ denotes the relative error between the estimated lower fitting limit d_L_ and the transition diameter d_c_ identified under threshold β. Both d_L_ and d_c_ are expressed in mm.

## Data Availability

The original contributions presented in this study are included in the article/Supplementary Material. Further inquiries can be directed to the corresponding authors.

## Supplementary Materials

The following supporting information can be downloaded at: https://www.mdpi.com/article/10.3390/ma19122675/s1, Table S1. complete gradation compositions of the progressively truncated specimens.

## Abbreviations

Abbreviation	Definition
PSD	Particle-size distribution
DASR	Dominant aggregate size range
TBM	Tunnel boring machine
