# Supplementary material for: Packing Densification Response–Constrained Fractal Characterization and Compaction Performance Evaluation of Widely Graded Granular Materials
Source: Materials (Basel). 2026 Jun 22;19(12):2675. doi: 10.3390/ma19122675 (PMC13304443; doi:10.3390/ma19122675)
Supplement: Supplementary file 1 [file materials-19-02675-s001.zip › materials-4344875-supplementary.pdf]

materials

MDPI

Supplementary Materials

Table S1. complete gradation compositions of the progressively truncated specimens.

		Percent passing (%)									
**Sample No.**	**Sieve size (mm)**	0.075	0.25	0.5	1	2	5	10	20	40	60
A-0	n=0.35	9.64	14.69	18.72	23.86	30.41	41.91	53.41	68.08	86.77	100
A-1		—	14.69	18.72	23.86	30.41	41.91	53.41	68.08	86.77	100
A-2		—	—	18.72	23.86	30.41	41.91	53.41	68.08	86.77	100
A-3		—	—	—	23.86	30.41	41.91	53.41	68.08	86.77	100
A-4		—	—	—	—	30.41	41.91	53.41	68.08	86.77	100
A-5		—	—	—	—	—	41.91	53.41	68.08	86.77	100
A-6		—	—	—	—	—	—	53.41	68.08	86.77	100
B-0	n=0.55	2.53	4.91	7.19	10.52	15.40	25.49	37.33	54.65	80.01	100
B-1		—	4.91	7.19	10.52	15.40	25.49	37.33	54.65	80.01	100
B-2		—	—	7.19	10.52	15.40	25.49	37.33	54.65	80.01	100
B-3		—	—	—	10.52	15.40	25.49	37.33	54.65	80.01	100
B-4		—	—	—	—	15.40	25.49	37.33	54.65	80.01	100
B-5		—	—	—	—	—	25.49	37.33	54.65	80.01	100
B-6		—	—	—	—	—	—	37.33	54.65	80.01	100
C-0	n=0.75	0.66	1.64	2.76	4.64	7.80	15.51	26.08	43.87	73.78	100
C-1		—	1.64	2.76	4.64	7.80	15.51	26.08	43.87	73.78	100
C-2		—	—	2.76	4.64	7.80	15.51	26.08	43.87	73.78	100
C-3		—	—	—	4.64	7.80	15.51	26.08	43.87	73.78	100
C-4		—	—	—	—	7.80	15.51	26.08	43.87	73.78	100
C-5		—	—	—	—	—	15.51	26.08	43.87	73.78	100
C-6		—	—	—	—	—	—	26.08	43.87	73.78	100

https://doi.org/10.3390/ma19122675
